# Phytochemical Investigation and Antioxidant Activity of *Globularia alypum* L.

**DOI:** 10.3390/molecules26030759

**Published:** 2021-02-02

**Authors:** Fadoua Asraoui, Ayoub Kounnoun, Hafssa El Cadi, Francesco Cacciola, Yassine Oulad El Majdoub, Filippo Alibrando, Filippo Mandolfino, Paola Dugo, Luigi Mondello, Adnane Louajri

**Affiliations:** 1 Laboratory of Applied Biology and Pathology, Department of Biology, Faculty of Sciences of Tetouan, Abd Al-Malek Essaadi University, Tetouan 93000, Morocco; a.kounnoun@gmail.com (A.K.); alouajri@hotmail.com (A.L.); 2 Laboratory of Valorization of Resources and Chemical Engineering, Department of Chemistry, Abdelmalek Essaadi University, Tangier 90000, Morocco; hafssa.elcadi@yahoo.fr; 3 Department of Biomedical, Dental, Morphological and Functional Imaging Sciences, University of Messina, 98125 Messina, Italy; 4 Department of Chemical Biological, Pharmaceutical and Environmental Sciences, University of Messina, 98168 Messina, Italy; youladelmajdoub@unime.it (Y.O.E.M.); fmandolfino@libero.it (F.M.); pdugo@unime.it (P.D.); lmondello@unime.it (L.M.); 5 Chromaleont s.r.l., c/o Department of Chemical, Biological, Pharmaceutical and Environmental Sciences, University of Messina, 98168 Messina, Italy; filippo.alibrando@chromaleont.it; 6 Foundation A. Imbesi c/o, University of Messina, 98122 Messina, Italy; 7 Department of Sciences and Technologies for Human and Environment, University Campus Bio-Medico of Rome, 00128 Rome, Italy; 8 BeSep s.r.l., c/o Department of Chemical, Biological, Pharmaceutical and Environmental Sciences, University of Messina, 98168 Messina, Italy

**Keywords:** *Globularia alypum* L., HPLC-DAD/ESI-MS, GC-MS, phenolic compounds, flavonoids, antioxidant activity

## Abstract

The Moroccan flora is rich in medicinal plants that are commonly used in folk medicine for the treatment of various diseases. The present study was designed to investigate the total phenolic and flavonoid contents, as well as the antioxidant properties of leaves extracts from *Globularia alypum* L. colected from the Taza region in northeast Morocco. Additionally, the individual phenolics and volatiles of the extracts were also evaluated. The organic extracts of this plant were obtained by Soxhlet extraction using two different solvents, namely ethyl acetate and chloroform. The antioxidant capacity of leaves extracts was measured using DPPH, ABTS and FRAP assays; the phenolic profile was determined by HPLC-DAD/ESI-MS analysis, whereas the volatile composition was elucidated by GC-MS. The ethyl acetate extract analysis showed a total of 20 phenolic compounds and the determination of phenolic contents showed a significant value of 56.5 ± 0.61 µg GAE/mg of extract in comparison with the chloroform extract (18.9 ± 0.48 µg GAE/mg of extract). Also, the determination of the flavonoid contents revealed that the ethyl acetate extract contained the highest value (30.2 ± 0.55 µg CE/mg of extract) in comparison with the chloroform extract (18.0 ± 0.36 µg CE/mg of extract). Concerning the antioxidant properties, interesting values were attained for the ethyl acetate extract which exhibited higher antioxidant activity, namely IC_50_ = 12.3 ± 3.83 µg/mL and IC50 = 37.0 ± 2.45 µg/mL for the DPPH and ABTS assays, respectively, whereas a value of 531.1 ± 17.08 (mg AAE/g DW) was obtained for the FRAP assay. Concerning the volatile profile, a total of 73 compounds were positively detected and among them *n*-hexadecanoic acid (13.5%) was the most abundant one. The results achieved confirm the important role of this plant as a source of natural antioxidants.

## 1. Introduction

Since ancient times medicinal plants have been used as a treatment to cure several diseases thanks to their curative properties. Nowadays, despite the development of modern medicine and the effectiveness of synthetic drugs in treating different ailments, many people choose to use traditional remedies, especially for their fewer side effects compared to chemical drugs. Medicinal plants are able to produce a large number of diverse bioactive compounds, particularly secondary metabolites. For this reason, extensive studies using different plant extracts have been reported by several scientists, to investigate the antibacterial, anti-inflammatory, analgesic, antioxidant and many other medicinal values of these extracts [1,2,3].

Reactive oxygen species (ROS) serve as cell signaling molecules for normal biologic processes; in general, the disproportion between production of the ROS and the biological system ability of the normal detoxification leads to oxidative stress. These disturbances in the normal redox state of cells can cause toxic effects through the production of free radicals that react with cellular constituents and caused severe damage to the cells [4,5,6]. Free radical mechanisms have been involved in the pathology of several diseases including diabetes, rheumatoid arthritis, cancer, neurodegenerative diseases, atherosclerosis, etc. [7]. Antioxidants can be defined as any compounds able to react with free radicals by neutralizing them to non-radical products and, consequently, these compounds can be used to stop or minimize the deleterious effects caused by free radicals in the human body [8]. Since the discovery of antioxidants, many types of analysis have been done to determine these substances, in terms of benefits and risks [9].

Various aromatic and medicinal plants have been reported as an important source of natural antioxidants due to the activity of their secondary metabolites. To this regard, several studies have revealed that antioxidant compounds play important biological properties that might be used for food conservation, cosmetics and pharmaceutical products, alternative medicine and natural therapies [10].

*Globularia alypum* L. (*G. A*.) is a wild perennial shrub, belonging to the Plantaginaceae family which is found throughout the Mediterranean area. The plant, locally named “Ain Larnab” or “Tasselgha”, is known for a variety of purposes in the Moroccan traditional medicine [11]. Leaves of *G. A.* are traditionally used as an antidiabetic agent, laxative, stomachic, and purgative [12]. In Algeria, this plant has been used also in the treatment of urinary incontinence and skin problems, such as eczemas, according to an ethnobotanical survey, which showed that *G. A.* is one of the most important medicinal plants used in traditional remedies by Algerian people [13,14].

The aim of the present study was to determine the total phenolic and flavonoid contents in two different extracts of *G. A.* and the investigation of the antioxidant activities of these extracts by using three different methods, namely free radical scavenging assays (ABTS and DPPH tests), and reducing power assays (FRAP). In addition, a chemical determination of phenolic compounds and volatile profile of *G. A.* extract was determinated by the high-performance liquid chromatography coupled to photodiode array and electrospray ionization mass spectrometry (HPLC-DAD/ESI-MS) and gas chromatography coupled to MS detection.

## 2. Results and Discussion

### 2.1. Determination of Phenolic Compounds

The total phenolic content (TPC) is reported as gallic acid equivalents (GAE) (Y = 0.253x + 0.23, R^2^ = 0.999), and the obtained results showed that the highest TPC with (56.5 ± 0.61 µg GAE/mg of extract), was found in the ethyl acetate extract, while the chloroform extract showed the lowest value with (18.9 ± 0.48 µg GAE/mg of extract), (Table 1). The total flavonoid content (TFC) is reported as catechin equivalents (CE) (Y = 0.0054x + 0.1506, R^2^ = 0.998), and also in this case the ethyl acetate extract yielded the highest flavonoid content (30.2 ± 0.55 µg CE/mg of extract) when compared to the chloroform extract which presents a value of (18.0 ± 0.36 µg CE/mg of extract). By comparison of these results with the available literature on the same species, the obtained values are considerably higher than those reported by Athmouni et al. [15] reported that the values of TPC and TFC in leaves extracts of Tunisian G.A were 8.04 mg GAE/mg DW and 0.67 mg CE/mg DW, respectively. The same consideration can be done when comparing our values with the work by Chograni et al. [16] who determined lower content of TPC (22.30 mg GAE/g DW) and TFC content (4.72 mg RE/g D.W) of the methanolic leaves extracts of *G. A.* (Korbous Jebel Mountain, Tunisia). Also, El khantouch et al.’s study [17] reported lower TFC content (8.96 mg QE/g DW) for the hydro-acetate extract, as well the Djeridane et al.’s study [18] which carried out in Algeria, reported a value of 21.54 mg GAE/g DW and 4.54 mg RE/g DW of TPC and TFC, respectively. Further lower values were attained by Krimat et al. [19] who determined lower values of TPC content (25.38 mg GAE/g extract) and flavonoid content (3.76 mg QE/g extract) for the same extracts. On the other hand, a very recent study carried out by Tiss et al. [20] in Tunisia suggest significant values of TPC and TFC of 81.3 mg GAE/g and 61.2 mg RE/g, respectively.

A recent study by Taghzouti et al. [21] for Fès-Meknès region reported for the ethyl acetate extract a TPC value of 50.99 μg GAE/mg of extract, which is close to the one determined in the present study. However, the flavonoid content (8.96 μg QE/mg of extract) was lower, and this finding might be due to many factors, such as environmental conditions, extraction method and portion of the plant used.

On the basis of such considerations, such a species has a significant antioxidant potential, therefore, the analysis of individual phenolic acids and flavonoids, as well as antioxidant assays are important for a better understanding in a view of medicinal and/or pharmacological purposes.

### 2.2. Antioxidant Activity

**Figure 1:**
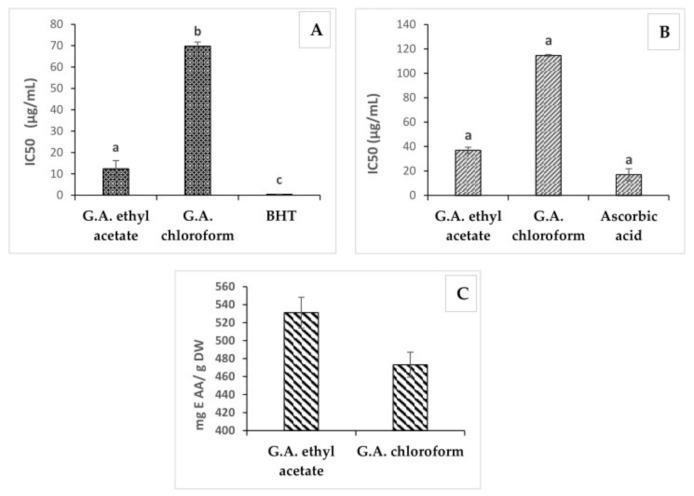
DPPH (**A**), ABTS (**B**) and FRAP (**C**) scavenging activity of ethyl acetate and chloroform extracts, BHT and Ascorbic acid of *G. A. Abbreviations:* FRAP, ferric reducing antioxidant power; DPPH, 2,2-diphenyl-1-picrylhydrazyl radical; ABTS, 2,2′-azino-bis (3-ethylbenzothiazoline-6-sulphonic) acid; IC50, Concentration of sample providing 50% inhibition.

The antioxidant properties of *G. A.* leaves were evaluated by DPPH, ABTS and FRAP assays; the results were expressed as IC_50_ values (Table 2). The antioxidant activity is based on the redox properties of the extracts which facilitate their activity as reducing agents; such ability is generally associated with the presence of reductants which exert antioxidant action through breaking the free radical chain by donating a hydrogen atom or preventing peroxide formation [22]. Medicinal plant tissues are rich in phenolic compounds and these compounds do have multiple biological effects including antioxidant. As shown in Table 2 the two extracts of *G. A.* showed significant antioxidant activity with significant variability between organic extracts and the used methods: the ethyl acetate extract was the most active in terms of antioxidant properties with values of IC50 = 12.3 ± 3.83 µg/mL and IC50 = 37.0 ± 2.45 µg/mL attained by DPPH and ABTS assays, respectively; a value of 531.1 ± 17.08 mg AAE/g DW was determined by FRAP assay. By comparison of the three methods used (Figure 1), the DPPH scavenging activity assay was the most sensitive one in terms of IC50. The ethyl acetate extract which contained the highest level of phenolic compounds and flavonoids yielded the smallest IC50 value, in agreement with previous studies [19,20,21,22,23]. Interestingly, DPPH and ABTS antioxidant activity is two-fold higher than the one described by Feriani et al. [24]. In addition, many studies proved that *G. A.* plant is rich in phenolic compounds, which are responsible for many activities including antioxidant, anticancer and anti-inflammatory activities [21,25,26]. 

### 2.3. GC-MS Analyses

**Figure 2:**
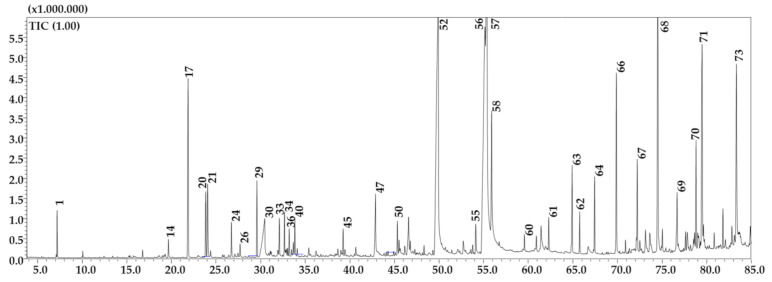
GC-MS profile of the *n*-hexane fraction of G.A. Main peaks are labeled. Peak assignment as in Table 3.

The attained results of the GC-MS analysis of the *n*-hexane fraction of *G. A.* showed the presence of lipids, alkanes, alcohols, terpenoids, etc. (Figure 2, Table 3), A total of 73 compounds were positively detected and among them *n*-hexadecanoic acid (13.5%) was the most abundant one, followed by oleic acid (12.98%) and linoleic acid (11.58%). The % of similarity ranged from 89 to 98%.

### 2.4. Phytochemical Profile by HPLC-DAD-ESI/MS

**Figure 3:**
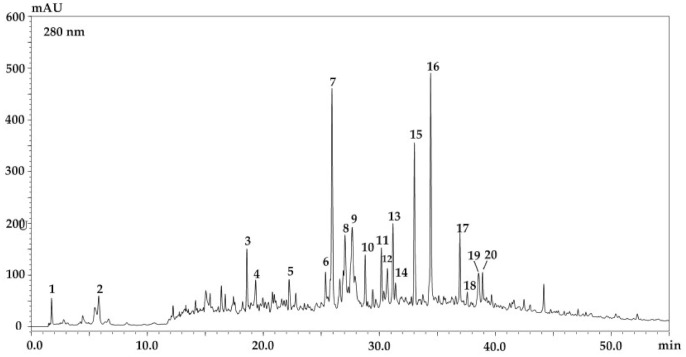
Phenolic profile of the ethyl acetate extract of *G. A.* at 280 nm.

In order to provide a phytochemical characterization of *G. A.* an HPLC-DAD-ESI/MS system was employed. Figure 3 and Table 4 report the phenolic content of the ethyl acetate extract which turned out to be the most complex one. A total of 20 compounds were detected and tentatively identified based on their retention times, MS data and comparison together with the information previously reported in the literature [24,25,26,27,28]. Three different phenolic compounds classes were present in the ethyl acetate extract namely phenolic acids (quinic acid, gallic acid, and gallic acid ethyl ether), flavonoids (derivatives of quercetin and kaempferol) and several iridoids. The content of phenolic acids and flavonoids were evaluated except for the iridoids. The whole content was as high as 46.6 ± 0.36 mg/g (*w/w*) (DW). The most abundant compound was represented by quercetin-rhamnoside 14.5 ± 1.20 mg/g (*w/w*) (DW), followed by kaempferol-derivative 10.8 ± 0.50 mg/g (*w/w*) (DW), which explain the agreement between the obtained results and those on the antioxidant activity and the TPC. The detected compounds have never been reported before as constituents of Moroccan *G. A.* [24,25,26,27,28,29].

## 3. Materials and Methods

### 3.1. Samples

*Globularia alypum* L. was collected from Taza region in Morocco, in the spring season between March and April 2018; plant identification was carried by Professor Mohamed El Kadiri, a botanist of the Abd Al-Malek Essaadi University (Tetouan, Morocco) and deposited in the herbarium of the Faculty with a voucher specimen code GA-LABP01. The fresh leaves of *G. A.* were dried on air at room temperature in shade approximately for 2 weeks. Once the leaves were well dried, they were powdered and stocked in glass bottle in the dark for further analyses.

### 3.2. Chemical Reagents and Solvents

2,2-Diphenyl-1-picrylhydrazyl (DPPH), 2,2′-azino-bis-(3-ethylbenzothiazoline-6-sulfonic) acid (ABTS), L-ascorbic acid, and butylated hydroxytoluene (BHT), were purchased from Sigma (St. Louis, MO, USA). Folin-Ciocalteu phenol reagent and standards (gallic acid, kaempferol and quercetin) were obtained from Merck Life Science (Merck KGaA, Darmstadt, Germany). LC-MS grade methanol, acetonitrile, acetic acid, acetone and water were also purchased from Merck Life Science. All other chemicals were of analytical grade and obtained from Sigma.

### 3.3. Preparation of Crude Extracts

The extraction of samples was conducted by Soxhlet apparatus with chloroform, and ethyl acetate, in order to get two extracts with different polarities. 50 g of dried leaves of *G. A.* were rigorously extracted with 250 mL of each solvent, then the obtained extracts were concentrated and free of solvent under reduce pressure, using rotary evaporator. At the end of the extraction operation two crude extracts were obtained and were subsequently weighed to calculate the yield of the extraction for each solvent and stored in a refrigerator (4 °C) in airtight bottles until used for further analysis.

For GC-MS analysis, five grams of plant powder was defatted three times in 50 mL of *n*-hexane, and the extraction was performed by sonication in an ultrasound bath (130 kHz) for 45 min. After centrifugation for 5 min, the supernatant was filtered through a paper filter, dried with rotary evaporator, and reconstituted with *n*-hexane, prior to GC-MS analysis.

### 3.4. Analysis and Quantification of Phenolic Contents

TPC of *G. A.* leaves extracts was determined by Folin-Ciocalteu colorimetric method as described by Singleton et al. 1965 [30,31] with slight modification. Briefly, 100 µL of the diluted extracts, 500 µL of the Folin-Ciocalteu reagent, 400 µL of 7.5% saturated aqueous sodium carbonate (Na_2_CO_3_), were mixed; the mixture was then thoroughly homogenized and incubated for 30 min at 40 °C. After incubation, the absorbance of each sample was measured at 765 nm against methanol as blank and the TPC was calculated by using the gallic acid calibration curve. All assays were performed in triplicate. The TPC was expressed as µg GAE/mg of extract.

TFC of *G. A.* leaves extracts was determined using the aluminum chloride colorimetric method, according to the protocol described by Dewanto et al. [32]. Briefly, 250 µL of each plant extract was mixed with 1250 µL of distilled water and subsequently with sodium nitrite solution (5% NaNO_2_); the mixture was incubated for 6 min at room temperature. Thereafter, 150 µL of aluminum trichloride solution (10% AlCl_3_) were added and then allowed to stand for 6 min. Afterwards, 500 µL of sodium hydroxide solution (4%NaOH) were dissolved in 275 µL of distilled water. Then, the mixture was thoroughly mixed and allowed to stand for 30 min at room temperature. The absorbance was measured at 510 nm *versus* reagent blank containing methanol instead of the sample. Catechin was used as a standard compound for the quantification of total flavonoids. All trials were performed in triplicate. Results were expressed as µg of catechin equivalents per mg DW extracts (µg CE/mg).

### 3.5. Determination of Antioxidant Activity

The organic extracts of *G. A.* leaves were subjected to screening for their possible antioxidant activity by three test systems namely DPPH, ABTS and FRAP methods.

#### 3.5.1. 2,2-Diphenyl-1-picrylhydrazyl Radical (DPPH) Free Radical-Scavenging Assay

The radical scavenging activities of the extracts of *G. A.* were measured using the stable free radical DPPH(2,2-diphenyl-1-picrylhydrazyl) according to a slightly modified method by Hatano et al. [33]. DPPH methanolic solution (500 µL, 0.2 mM) was added to leaves of *G. A.* (2 mL, 2.5–100 µg/mL). The mixture was vortexed thoroughly and after 30 min incubation time in darkness and at room temperature, the absorbance was measured at 517 nm with a blank containing DPPH and methanol. The butylated hydroxytoluene (BHT) was used as a positive control and the DPPH radical scavenging activity was calculated according to the equation:

DPPH radical scavenging activity I (%) = (A blank − A sample)/A blank × 100(1)

The IC_50_ of DPPH radical was calculated from the line regression of the percentage of remaining DPPH radical against the sample concentration.

#### 3.5.2. 2,2′-Azino-bis(3-ethylbenzothiazoline-6-sulphonic) Acid (ABTS) Radical Scavenging Activity

ABTS radical scavenging activity was determined according the method of Re et al. [34]. The ABTS stock solution was produced by reacting ABTS aqueous solution (7 mM) with 2.45 mM aqueous solution of potassium persulfate, and allowed the mixture to react in the dark at room temperature for 12–16 h; then the ABTS^+^ stock solution was diluted with methanol to an absorbance of 0.7 ± 0.02, at 734 nm. 15 µL of the test sample was mixed with 185 µL of ABTS^+^ diluted solution, and the mixture was incubated for 10 min. The results were expressed as IC_50_ (µg/mL) and the percentage of inhibition was calculated according to the following equation:

ABTS Scavenging effect % = [(A_0_ − A_1_)/A_0_] × 100 (2)

where: A_0_ is the absorbance of the control, and A_1_ is the absorbance of the sample.

#### 3.5.3. Ferric Reducing Power Determination

The reducing power of extracts was determined according to the method reported by Oyaizu [35] with minor modifications: a volume of 1 mL of test sample solution (10 µg/mL) was mixed with 2.5 mL of potassium ferricyanide (1% *w*/*v*), and 2.5 mL of phosphate buffer (0.2 M, pH 6.6). Then the mixture was incubated for 20 min at 50 °C, and 2.5 mL of trichloroacetic acid (10%) were added. After centrifugation of the mixture for 10 min at 3000 rpm, 2.5 mL were collected from the upper layer, mixed with 2.5 mL of distilled water, and 0.5 mL of FeCl_3_ (0.1%). Ascorbic acid (10 µg/mL) was used as a standard for the preparation of the calibration curve, and the absorbance was measured at 700 nm by a spectrophotometer. Results were expressed as mg of ascorbic acid equivalents per mg AAE/g DW. All tests were performed in triplicate.

### 3.6. GC-MS

The analysis of the volatile fraction was performed on a GC-MS-QP2020 system (Shimadzu, Kyoto, Japan) with an “AOC-20i” system auto-injector. The analyses were carried out on an SLB-5ms column (30 m in length × 0.25 mm in diameter × 0.25 μm in thickness of film, Merck KGaA). The initial temperature was set at 50 °C, afterwards increased up to 350 °C (increase rate: 3 °C/min; holding time: 5 min). GC-MS parameters were as follows: injection temperature: 280 °C; injection volume: 1.0 μL (split ratio: 10:1); pure helium gas (99.9%); linear velocity: 30.0 cm/s; Inlet pressure: 26.7 KPa. EI source temperature: 220 °C; Interface temperature: 250 °C. The acquisition of MS spectra was carried out in full scan mode, in the mass range of 40–660 *m*/*z*, with an event time of 0.2 s. Relative quantity of the chemical compounds present in each sample was expressed as percentage based on peak area produced in the GC chromatogram. 

Compounds were identified by using the “FFNSC 4.0” (Shimadzu Europa GmbH, Duisburg, Germany), and “W11N17” (Wiley11-Nist17, Wiley, Hoboken, NJ, USA; Mass Finder 3). Each compound was identified applying a MS similarity match and an LRI filter. Linear retention indices (LRI) were calculated by using a C7-C40 saturated alkanes reference mixture (49452-U, MerckKGaA). Data files were collected and processed by using “GCMS Solution” software, ver. 4.50 (Shimadzu).

### 3.7. HPLC-DAD/ESI-MS

LC analyses were carried out on a Shimadzu liquid chromatography system (Kyoto, Japan) consisting of a CBM-20A controller, two LC-20AD dual-plunger parallel-flow pumps, a DGU-20A5R degasser, a SIL-20AC autosampler, an SPD-M30A photo diode array detector and an LCMS-2020 mass spectrometer, through an ESI source (Shimadzu, Kyoto, Japan).

Chromatographic separations were performed on 150 × 4.6 mm; 2.7 µm Ascentis Express RP C18 column (Merck Life Science, Merck KGaA, Darmstadt, Germany) [36,37,38]. The mobile phase was composed of two solvents: water/acetic acid (99.85/0.15 *v/v*, solvent A) and acetonitrile/acetic acid (99.85/0.15 *v/v*, solvent B), The flow rate was fixed at 1 mL/min under gradient elution: 0–5 min, 5% B, 5–15 min, 10% B, 15–30 min, 20% B, 30–60 min, 50% B, 60 min, 100% B. DAD detection was applied in the range of λ = 200–400 nm and a wavelength of 280 nm was monitored (sampling frequency: 40.0 Hz, time constant: 0.08 s). MS conditions were as follows: scan range and scan speed were set at *m*/*z* 100–800 and 2500 u s^−1^, respectively, event time: 0.3 s, nebulizing gas (N_2_) flow rate: 1.5 L min^−1^, drying gas (N_2_) flow rate: 15 L min^−1^, interface temperature: 350 °C, heat block temperature: 300 °C, DL (desolvation line) temperature: 300 °C, DL voltage: 1 V, interface voltage: −4.5 kV. Calibration curves (R^2^ ≥ 0.997) of three phenolic standards were used for the quantification of the ethyl acetate extract.

### 3.8. Statistical Analysis

All data were analysed using IBM SPSS Statistics for Windows, version 21 (IBM Corp., Armonk, N.Y., USA). The experiments were carried out in triplicates and the results are expressed as the average of the three measurements ± SD. The comparison of means between groups was performed with one-way analysis of variance (ANOVA) followed by Tukey test. Differences were considered significant when *p* < 0.05.

## 4. Conclusions

In this contribution, total phenolic and total flavonoid contents as well as the antioxidant capacity evaluated by ABTS, FRAP and DPPH assays of ethyl acetate and chloroform leaves extracts of *Globularia alypum*, L. grown in Morocco, were investigated. The phenolic profile revealed a total of 20 compounds including phenolic acids, flavonoids and iridoids in the ethyl acetate extract, whereas the volatile profile showed a total of up to 73 compounds belonging to different chemical classes. The results achieved are very promising and if supported by “in vivo” studies might propose such a species to be used for therapeutic purposes.

## Figures and Tables

**Table 1:** Phytochemical contents of *G. A.* leaves extracts.

Plant Extracts	Polyphenols (µg GAE/mg of Extract)	Flavonoids (µg CE/mg of Extract)
Ethyl acetate	56.5 ± 0.61	30.2 ± 0.55
Chloroform	18.9 ± 0.48	18.0 ± 0.36

µg GAE/mg extract: µg gallic acid equivalents per mg of extract; µg CE/mg extract: μg of catechin equivalents per mg of extract.

**Table 2:** Antioxidant activity of *G. A.* extracts.

	Antioxidant Properties (Mean IC_50_ Value µg/mL ± Standard Deviation)		
Plant Extracts	DPPH	ABTS	Reducing Power (mg AAE/g DW)
Ethyl acetate	12.3 ± 3.83	37.0 ± 2.45	531.1 ± 1.08
Chloroform	69.8 ± 1.89	114.6 ± 0.63	473.2 ± 1.88

**Table 3:** List of compounds identified in *G.A.* by GC-MS.

Peak	Compound	Similarity %	LRI (lib)	LRI (exp)	Area %	Library
1	Pimelic ketone	98	901	898	0.45	FFNSC 4.0
2	*n-*Hexanoic acid	95	997	975	0.01	FFNSC 4.0
3	*p-*Cymene	90	1025	1025	0.01	FFNSC 4.0
4	Benzyl alcohol	96	1040	1036	0.02	FFNSC 4.0
5	γ-Terpinene	93	1058	1058	0.02	FFNSC 4.0
6	(Z)-Sabinene hydrate	91	1069	1071	0.01	FFNSC 4.0
7	Linalool	94	1101	1100	0.03	FFNSC 4.0
8	(E)-Sabinene hydrate	92	1099	1102	0.02	FFNSC 4.0
9	*n-*Nonanal	92	1107	1106	0.01	FFNSC 4.0
10	Maltol	91	1108	1111	0.03	FFNSC 4.0
11	Borneol	95	1173	1174	0.03	FFNSC 4.0
12	Terpinen-4-ol	92	1184	1182	0.03	FFNSC 4.0
13	2-(2-butoxyethoxy)-Ethanol	95	1192	1186	0.03	W11N17
14	*p-*Menth-3-en-7-al	91	1195	1197	0.24	FFNSC 4.0
15	*n-*Decanal	90	1208	1207	0.02	FFNSC 4.0
16	3-ethyl-4-methyl-1H-Pyrrole-2,5-dione	90	1234	1235	0.01	W11N17
17	Cuminaldehyde	95	1243	1246	2.75	FFNSC 4.0
18	*n-*Nonanoic acid	93	1289	1269	0.03	FFNSC 4.0
19	Phellandral	92	1277	1280	0.01	FFNSC 4.0
20	α-Terpinen-7-al	98	1287	1289	0.91	FFNSC 4.0
21	γ-Terpinen-7-al	91	1292	1294	1.21	FFNSC 4.0
22	*p-*Mentha-1,4-dien-7-ol	91	1327	1332	0.04	FFNSC 4.0
23	δ-Elemene	94	1335	1336	0.03	FFNSC 4.0
24	Eugenol	95	1357	1355	0.48	FFNSC 4.0
25	2,6-dimethyl-2,7-Octadiene-1,6-diol	93	1367	1364	0.05	W11N17
26	α-Copaene	96	1375	1377	0.16	FFNSC 4.0
27	β-Elemene	90	1390	1391	0.03	FFNSC 4.0
28	Vanillin	90	1394	1399	0.01	FFNSC 4.0
29	(E)-Caryophyllene	96	1424	1421	1.08	FFNSC 4.0
30	(E)-Cinnamic acid	96	1454	1443	1.52	FFNSC 4.0
31	(E)-, β-Farnesene	92	1452	1453	0.01	FFNSC 4.0
32	α-Humulene	95	1454	1457	0.06	FFNSC 4.0
33	α-Curcumene	91	1480	1482	0.51	FFNSC 4.0
34	α-Zingiberene	91	1496	1496	0.61	FFNSC 4.0
35	(E,E)-, α-Farnesene	96	1504	1505	0.09	FFNSC 4.0
36	β-Bisabolene	94	1508	1509	0.37	FFNSC 4.0
37	γ-Cadinene	95	1512	1516	0.09	FFNSC 4.0
38	Cubebol	89	1419	1518	0.02	FFNSC 4.0
39	δ-Cadinene	93	1518	1521	0.22	FFNSC 4.0
40	β-Sesquiphellandrene	94	1523	1525	0.55	FFNSC 4.0
41	(R)-5,6,7,7a-Tetrahydro-4,4,7a-trimethyl- 2(4H)-benzofuranone	93	1525	1532	0.15	W11N17
42	*n*-Dodecanoic acid	95	1570	1565	0.17	FFNSC 4.0
43	Caryophyllene oxide	92	1587	1585	0.09	FFNSC 4.0
44	*n-*Hexadecane	93	1600	1599	0.04	FFNSC 4.0
45	*ar-*Tumerone	97	1668	1666	0.37	FFNSC 4.0
46	*n-*Heptadecane	91	1699	1700	0.05	FFNSC 4.0
47	*n-*Tetradecanoic acid	90	1773	1766	1.28	FFNSC 4.0
48	Octadec-1-ene	95	1793	1793	0.03	FFNSC 4.0
49	*n-*Octadecane	96	1800	1799	0.04	FFNSC 4.0
50	Neophytadiene	95	1836	1836	0.45	FFNSC 4.0
51	methyl-Hexadecanoate	93	1925	1926	0.12	FFNSC 4.0
52	*n-*Hexadecanoic acid	92	1977	1974	13.55	FFNSC 4.0
53	isopropyl-Hexadecanoate	93	2023	2023	0.04	FFNSC 4.0
54	*n-*Heptadecanoic acid	91	2065	2065	0.26	W11N17
55	Phytol	96	2111	2111	0.56	FFNSC 4.0
56	Linoleic acid	92	2144	2146	11.58	FFNSC 4.0
57	Oleic acid	90	2142	2153	12.98	FFNSC 4.0
58	Stearic acid	90	2165	2171	3.93	FFNSC 4.0
59	*n-*Docosane	95	2200	2200	0.05	FFNSC 4.0
60	*n-*Tricosane	94	2300	2300	0.2	FFNSC 4.0
61	*n-*Tetracosane	94	2400	2399	0.44	FFNSC 4.0
62	Behenyl alcohol	98	2493	2496	0.11	FFNSC 4.0
63	*n-*Pentacosane	93	2500	2500	1.23	FFNSC 4.0
64	*n-*Hexacosane	94	2600	2599	1.01	FFNSC 4.0
65	Octocrylene	92	2658	2654	0.03	FFNSC 4.0
66	*n-*Heptacosane	92	2700	2700	2.64	FFNSC 4.0
67	*n-*Octacosane	93	2800	2799	1.17	FFNSC 4.0
68	*n-*Nonacosane	90	2900	2900	4.2	FFNSC 4.0
69	*n-*Triacontane	91	3000	2999	0.84	FFNSC 4.0
70	*n-*Hentriacontane	90	3100	3100	1.59	FFNSC 4.0
71	Vitamin E	94	3138	3133	4.23	W11N17
72	*n-*Dotriacontane	91	3200	3199	0.22	FFNSC 4.0
73	γ-Sitosterol	95	3351	3325	4.13	W11N17
	**TOTAL IDENTIFIED**				**79.59**	

**Table 4:** Compounds detected in the ethyl acetate extract of *G. A.* by HPLC-DAD-ESI/MS.

Peak	t_R_ (min)	λ_MAX_ (nm)	*m*/*z*	Fragments	Tentative Identification	Detection	Concentration (mg/100 g)
1	1.74	240	191	-	Quinic acid	DAD/MS	-
2	5.81	270	169	-	Gallic acid	DAD/MS	4.3 ± 0.12
3	18.58	274	197	-	Gallic acid ethyl ester	DAD/MS	4.2 ± 0.20
4	19.33	272	373	392	Gardoside/geniposidic acid	DAD/MS	-
5	22.22	253–361	463	301	Quercetin glucoside	DAD/MS	4.7 ± 0.20
6	25.37	277	468	-	Unknown	-	-
7	25.92	274	489	-	Acetylbarlerin	DAD/MS	-
8	27.04	253–356	447	301	Quercetin rhamnoside	DAD/MS	14.5 ± 1.20
9	27.67	271–351	507	285	Kaempferol derivative	DAD/MS	10.8 ± 0.50
10	28.79	283	505	301	Quercetin acetyl hexoside	DAD/MS	0.3 ± 0.01
11	30.18	215–279	469	-	Unknown	DAD/MS	-
12	30.69	271	503	-	Serratoside	DAD/MS	-
13	31.18	268–350	477	-	Calceolarioside	DAD/MS	-
14	31.40	256–346	447	301	Quercetin glucoside	DAD/MS	7.8 ± 0.30
15	33.03	275	641	320	Unknown	-	-
16	34.44	277	517	-	Phellamurin	DAD/MS	-
17	36.96	221–283	525	-	Unknown	-	-
18	37.60	226–269	527	-	Globularioside/baldaccioside	DAD/MS	-
19	38.57	335–371	543	-	Unknown	DAD/MS	-
20	38.92	230–324	513	-	Unknown	DAD/MS	-

## Data Availability

Not applicable.

## Sample Availability

Samples of the compounds are not available from the authors.
